# COVID-19-related research in Africa: a cross-sectional review of the International Clinical Trial Registration Platform (ICTRP)

**DOI:** 10.1186/s13063-021-05621-x

**Published:** 2021-10-07

**Authors:** Bassey Edem, Victor Williams, Chukwuemeka Onwuchekwa, Ama Umesi, Marianne Calnan

**Affiliations:** 1 Vaccines and Immunity Theme, MRC Unit the Gambia at the London School of Hygiene and Tropical Medicine, Fajara, Gambia; 2 Unit of Epidemiology and Biostatistics, School of Public Health, Faculty of Health Sciences, University of the Witwatersrand, Johannesburg, South Africa; 3 Barcelona Institute for Global Health (ISGlobal), Barcelona, Spain; 4 University Research Co., LLC, Manila, Philippines

## Abstract

**Objective:**

The declaration of the coronavirus disease (COVID-19), a pandemic in early 2020, has seen an upsurge in research globally to fill gaps in the epidemiology of the SARS-CoV-2 virus impact on health care and clinical management, as well as possible prevention and treatment modalities. Published literature on the different types of COVID-19 research conducted globally is varied and is particularly limited in Africa. This study sets out to describe the COVID-19-related research registered and conducted on the African continent.

**Methods:**

This is a cross-sectional study of all COVID-19-related studies available in the WHO’s International Clinical Trials Registry Platform (ICTRP) repository. We extracted studies registered from March 1, 2020, to July 15, 2021. A descriptive analysis of the extracted data was performed, and the findings were presented.

**Results:**

At extraction, a total of 12,533 COVID-19-related studies were listed on the ICTRP portal. We included 9803 studies, after excluding 2060 duplicate records and 686 records without a site/country. While 9347 studies (96%) were conducted outside of Africa, only 456 studies (4%) were conducted in the African continent, of which 270 (59.2%) were interventional studies, and 184 (40.4%) were observational studies. About 80% of the studies were conducted in Egypt and South Africa, and most of these involved testing of drugs and biologicals.

**Conclusion:**

The African continent hosts considerably fewer COVID-19-related research compared to other parts of the world. This may have implications on scientific evidence available for implementing COVID-19 control efforts. There is, therefore, a need for local funding and ownership of research projects and north-south collaboration in research.

## Background

The World Health Organization’s declaration of the severe acute respiratory syndrome coronavirus-2 (SARS-CoV-2)-related COVID-19 as a pandemic in March 2020, resulted in the imposition of various restrictions globally, leading to a “shut down” of social and economic activities [1]. Consequently, several studies have been implemented globally to fill the knowledge gap on the epidemiology of this novel disease and develop and test safe and efficacious interventions such as vaccines and drugs. Indeed, this period has witnessed the most rapid implementation of studies on a public health condition, coupled with the equally rapid dissemination of evidence. The Cochrane group currently hosts a living systematic review of COVID-19 interventions with 3312 clinical trials on record as of August 14, 2021 (https://covid-nma.com/). Most reported clinical trials on COVID-19 are conducted in the USA, Europe, and China [2–4].

With the rapid turn-over of evidence on COVID-19, it has become progressively more challenging to keep track of what can only be described as a very “fluid” research landscape. It is vital to have an overarching view of what is being researched and on whom these studies are conducted. For Africa, a region that historically hosts the least clinical trials [5, 6], this is of particular importance in ensuring equity in the distribution of the COVID-19 research burden and access to interventions for the control and prevention of the pandemic.

Although only randomized trials require registration as per the International Committee of Medical Journal Editors (ICMJE) and WHO requirements [7, 8] to enhance transparency and minimize publication bias in clinical trials, there has been a call for the registration of observational studies for similar reasons [9, 10], and indeed, some observational studies are routinely registered in registers such as the ClinicalTrials.gov, albeit in numbers lower than randomized trials [11]. There is limited literature on the types of COVID-19 studies conducted in Africa. In this paper, we describe the characteristics of different registered COVID-19-related studies listed in the International Clinical Trials Registry Platform (ICTRP) that are ongoing in Africa.

## Methods

This research is a cross-sectional study of all COVID-19 studies in the WHO’s International Clinical Trial Registration Platform (ICTRP) International Clinical Trials Registry Platform (ICTRP) (who. int). The WHO provides a regularly updated repository of all COVID-19 studies registered in its network of partner registers.

The WHO’s clinical trials registry network was established in 2006 and comprised partner regional, or national clinical trial registers that upload information on the studies they hold at defined intervals. The portal provides a single, publicly accessible repository of clinical trials conducted globally. The WHO provides a separate, curated summary of global COVID-19 studies provided by partner registers in its network. We accessed the ICTRP on July 28, 2021, and extracted a listing of all COVID-19-related studies registered in the ICTRP from March 1, 2020, to the last update done on July 15, 2021, for analysis. We chose March 2020 as this was when COVID-19 was declared a pandemic, and countries were implementing various measures to adapt to the pandemic.

### Study variables

**Fig. 1:**
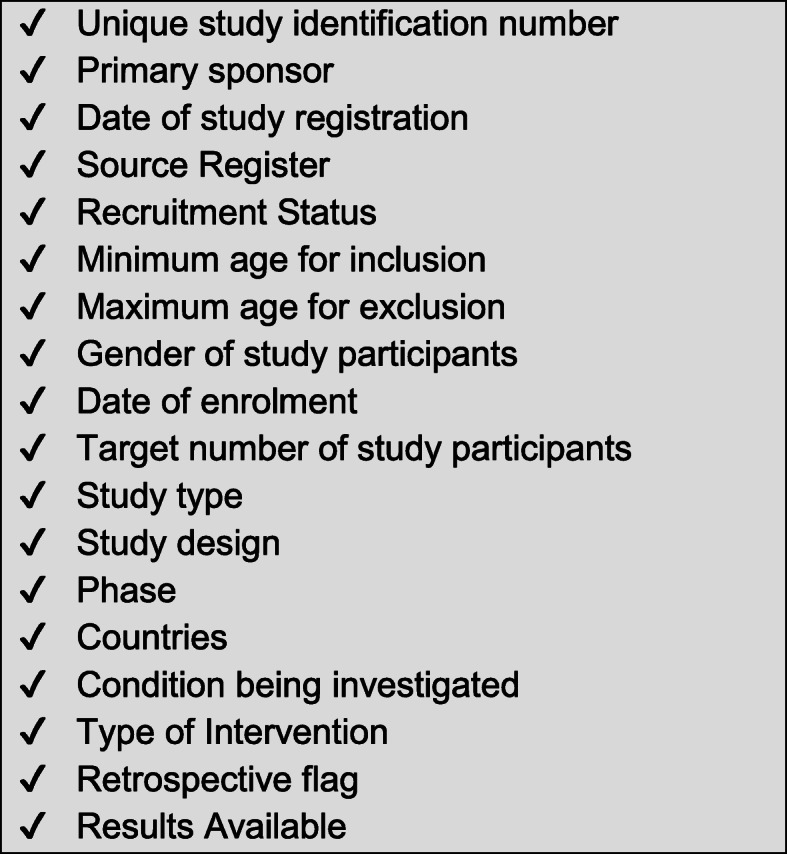
List of variables included in the study

The register included forty variables that described the studies listed. After a thorough review, the study team selected eighteen variables aligned with the study’s intended inclusion objectives. The first key variable was the study’s location as we sought to describe COVID-19-related research in Africa. Other vital variables included those that enabled unique identification of each study in the register, study title/objectives, study design, sample size, and target population. Additional variables were the study sponsor, recruitment status, phase of the study, the condition being investigated/specific intervention, the primary outcome, and results were available. The list of variables included in the study is outlined in Fig. 1.

### Definitions

The source register was defined as the primary register the research was documented before uploading to ICTRP. The different registers are defined in Table 1.

**Table 1:** Registers listed in the study

Register	Full name
ANZCTR	Australia New Zealand Clinical Trials Registry
CTRI	Clinical Trials Registry-India
ChiCTR	Chinese Clinical Trial Register
ClinicalTrials.gov	ClinicalTrials.gov
EU Clinical Trials Register	European Union Clinical Trials Register
GermanCTR	German Clinical Trials Register
ISRCTN	International Standard Randomised Controlled Trials Number
JPRN	Japan Primary Registries Network
PACTR	Pan African Clinical Trials Registry
RePEc	Research Papers in Economics
TCTR	Thai Clinical Trials Registry

### Data management

Data were extracted in .xls format and imported into Stata 15 (Stata Corp, College Station TX) for different data management procedures and descriptive analysis. Each study in the ICTRP portal had a unique code assigned to it by the original clinical trial register when the study was first registered. With this unique code as a unique identifier for each study, we used Stata to identify the duplicate studies. The duplicate studies identified were further examined manually to ensure other study parameters such as the date and time of upload, the study title, and the location the studies were conducted were the same before the duplicates were dropped. Studies without a country or location they were conducted were excluded.

A Stata code was used to extract and assign countries in a predefined continent to enable further description and analysis. Variables in the dataset such as country, study design, and type of intervention were entered as free text. These were extracted, manually examined, and categorized before analysis. For missing observations, casewise deletion was employed, and the data was presented.

### Data analysis

A descriptive method was adopted for this study using tables and figures to present findings from the analysis. Categorical variables were described using proportions, while the median (IQR) was used to describe continuous variables. If a particular variable was presented in a qualitative format, similar responses were grouped where possible and presented as proportions. We noted that there were single and multi-country studies. A code was used to identify any study which had an African country as either the only country or one of the countries in the study was being conducted. This enabled the identification of studies that had an African country as a study site in multi-country studies. These were sorted, and the frequency of each African country was identified. African countries with the highest frequencies were presented. Descriptive characteristics of studies within the register are summarized and shown in a table. Three figures describe and compare identified study characteristics.

## Results

**Fig. 2:**
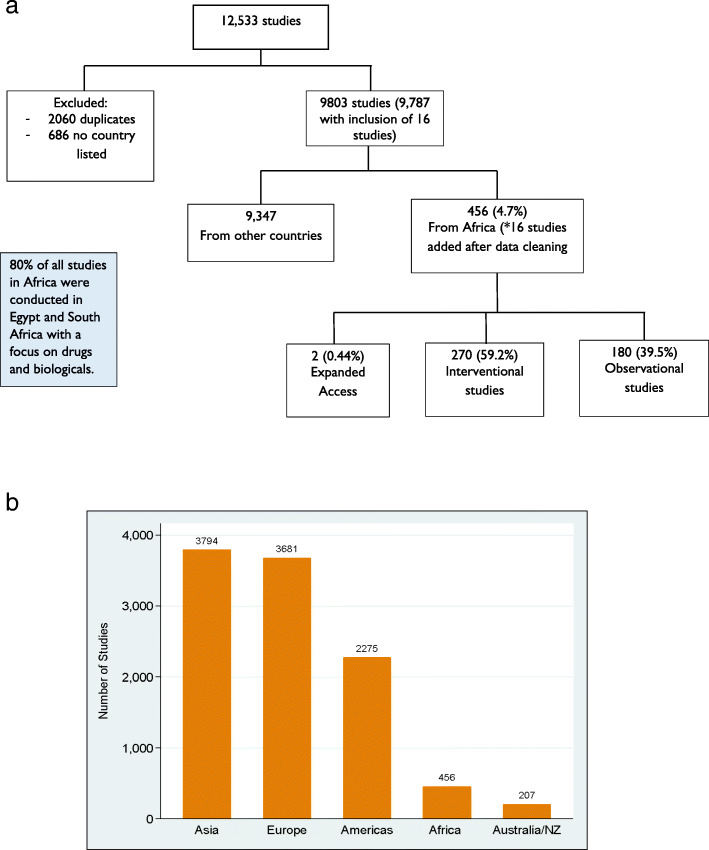
**a** Flowchart of studies. *16 studies added after data cleaning. See the “Results” section above for details. **b** Number of ongoing studies by continent

We identified 12,533 COVID-19-related studies in the register and included 9803 studies with complete information on the country in our analysis. During data cleaning, some [12] studies which had study sites in Africa were lost during the deduplication process. These were identified and included in the final dataset of studies conducted in Africa. Of the included studies, 456 (4.7%) were conducted in Africa (Fig. 2a, b). Most of the studies were conducted in Asia (3794 or 38.7%) and Europe (3681 or 37.5%). North and South America had 2275 registered studies (23.2%), and Australia/New Zealand had 207 studies (2.1%). However, 686 studies did not have countries or study locations indicated, 2060 studies were duplicate records, and these were excluded from further analysis (Fig. 2a).

**Fig. 3:**
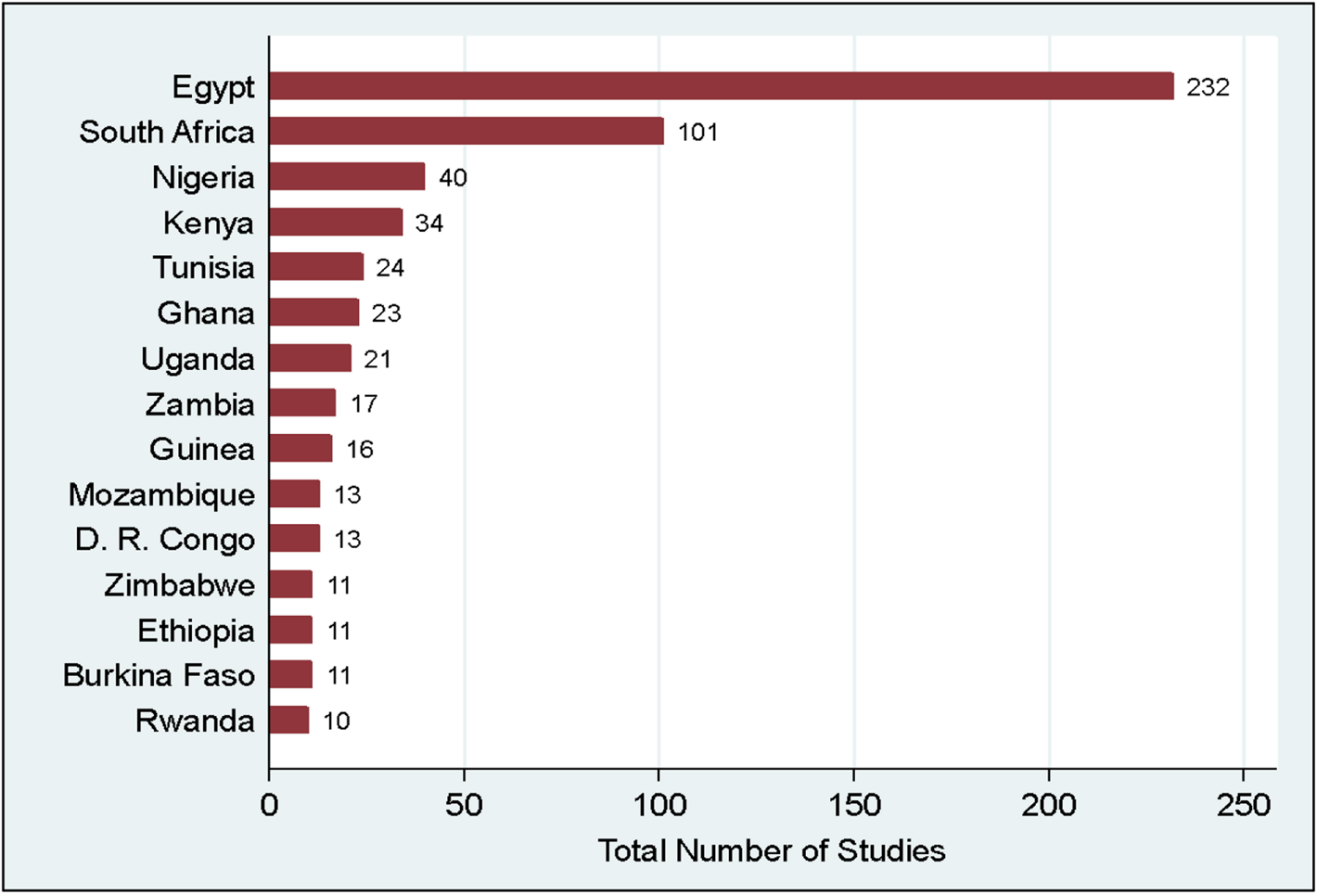
Number of ongoing studies in the first fifteen African countries. *The number of studies per country reflects the number of studies conducted within that country irrespective of other sites (multicenter)

The descriptive characteristics of the studies conducted in Africa are presented in Fig. 3 and Table 2. The African countries with the highest number of registered studies are shown in Fig. 3.

**Table 2:** Characteristics of studies conducted in Africa

Variable description	(***N*** = 456)
**Target size**	
Mean (SD)	6119 (36536)
Median (Q1, Q3)	264 (100, 1000)
**Source register**	
ANZCTR	4 (0.9%)
CTRI	15 (3.3%)
ChiCTR	2 (0.4%)
ClinicalTrials.gov	326 (71.5%)
EU Clinical Trials Register	22 (4.8%)
German Clinical Trials Register	3 (0.7%)
ISRCTN	18 (3.9%)
JPRN	5 (1.1%)
PACTR	53 (11.6%)
REPEC	7 (1.5%)
TCTR	1 (0.2%)
**Recruitment status**	
Authorized	20 (4.4%)
Not recruiting	214 (46.9%)
Recruiting	222 (48.7%)
**Study type**	
Expanded access	2 (0.4%)
Interventional study	270 (59.2%)
Observational study	184 (40.4%)
**Study design**	
Randomized	237 (52.0%)
Non-randomized	21 (4.6%)
Single group interventional study	12 (2.6%)
Blank	166 (36.4%)
Other	20 (4.4%)
**Phase**	
N/A	82 (28.0%)
Phase 0	1 (0.3%)
Phase 1	30 (10.2%)
Phase 1/phase 2	5 (1.7%)
Phase 2	40 (13.7%)
Phase 2/phase 3	27 (9.2%)
Phase 3	89 (30.4%)
Phase 4	19 (6.5%)
**Retrospective flag**	
No	76 (16.7%)
Yes	380 (83.3%)
**Sponsor**	
Other research/industry	202 (44.3%)
University	254 (55.7%)

Just more than half of the studies in Africa (232, representing 51.0%) were conducted in Egypt, followed by South Africa with 101 registered studies (24.1%). ClinicalTrials.gov was the commonest source register where studies were registered (71.5%), and most of the studies (59.2%) were interventional studies, with 184 observational studies (40.4%). The interventional studies included 237 randomized controlled trials, 21 non-randomized studies, and 12 single-group intervention studies. Most studies in Africa (55.7%) were sponsored by a university or a research institution affiliated with an academic institution.

### Types of COVID-19 research in Africa

**Fig. 4:**
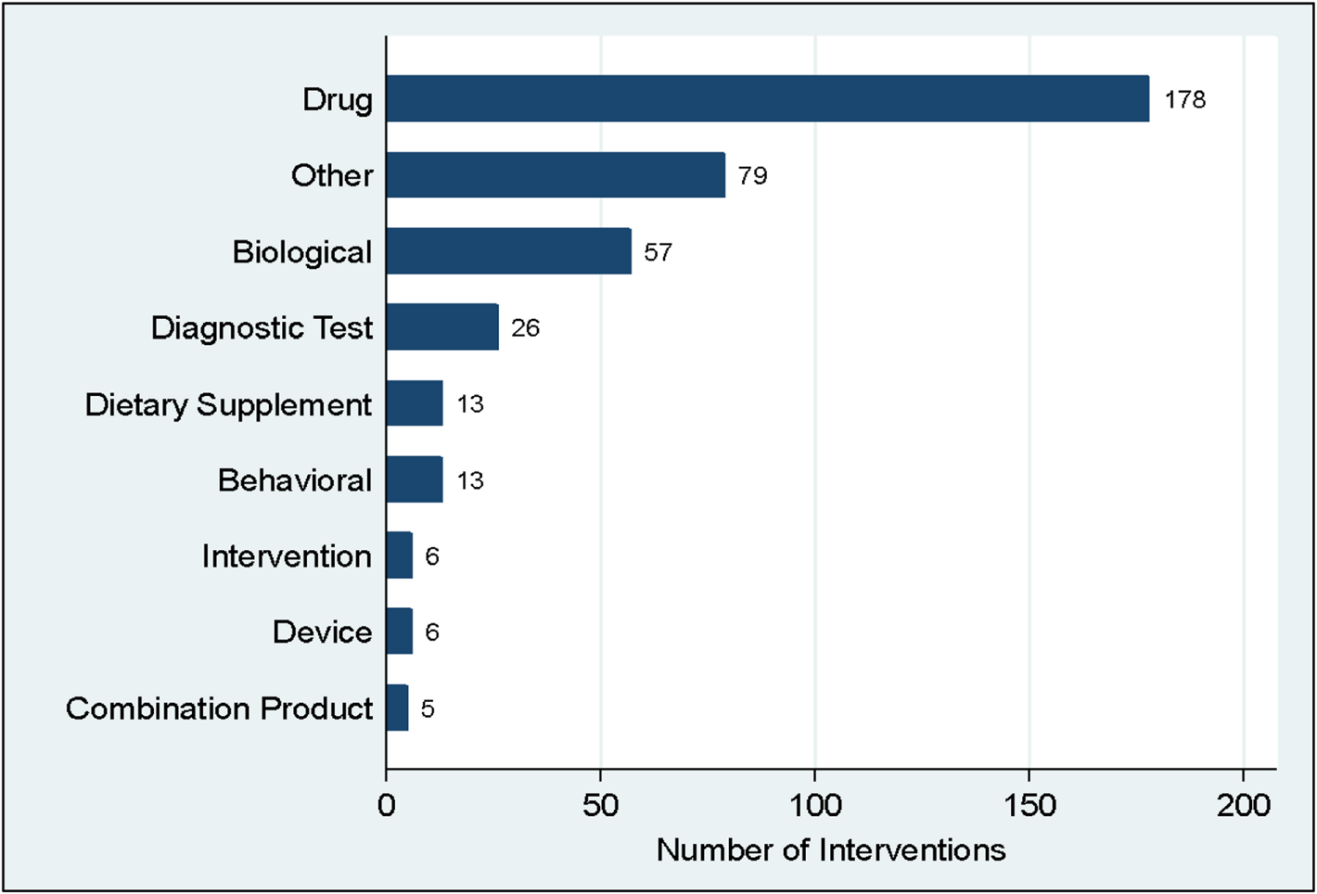
Types of COVID-19 research interventions in Africa

Three hundred eighty-three studies out of 456 in Africa had interventions documented (either interventional or observational design). Drug-related research was the most common type of research being conducted in Africa (46.6%) followed by studies on biological interventions (14.9%) and diagnostic tests (6.8%) (Fig. 4). Sub-analysis indicated the drugs being researched were predominantly the antimalarial medication hydroxychloroquine and its variants, Arthemeter, Artesunate, Paracetamol, Colchicine, Lopinavir/Ritonavir, and Atazanavir/Ritonavir. Biological interventions included the use of convalescent plasma or inducing the immune system using a vaccine—chadox, rabies, measles, mumps and rubella (MMR) vaccine, or low dose radiotherapy. We also included 79 studies where the intervention could not be clearly classified into one of the categories.

A sub-analysis of diagnostic test-related research included the use of tuberculin tests, whole-exome sequencing, real-time PCR, serological test, flow cytometry, and semen analysis. Interventions include studies that looked at the usual standard of care received by patients, enhanced care, and introduction of a new intervention to assess patient outcome. Further analysis of “Other” types of COVID-19 research indicates these were Pittsburgh Sleep Quality Index (PSQI) 1 (1%), usual care 1 (1%), prevention study 4 (5%), experimental study 5 (7%), training 5 [7], survey 21 (28%), and observational study 38 (51%).

## Discussion

We set out to describe the types of COVID-19 research conducted in Africa using data in the WHO’s International Clinical Trials Registry Platform (ICTRP) portal in this paper. The research priority areas have been identified by the World Health Organization (WHO) and include epidemiological studies of the virus to candidate vaccines research and development (R&D) [13]. Our results show that all forms of research on COVID-19 are being conducted in Africa. However, Africa hosts fewer COVID-19 trials than other regions, particularly in its size and population. This finding is consistent with data from the COVID-19 research funding tracker, which shows that of the 12,417 plus funded COVID-19 research projects, only 620 (4.9%) are conducted in Africa [14] and despite that, the location of 3% of these studies is unknown, this underscores the problem of minimal COVID-19 research in the continent.

There is considerable geographical disparity among COVID-19 research conducted in Africa, with three countries accounting for more than 80% of all registered studies. Although our results could not tell the funder of these studies, evidence showed that globally, funding of COVID-19 research is by European, Asian, and American institutions [14]. A study—not related to COVID-19 research—has highlighted the limited local funding for research in Africa, with three countries: Egypt, South Africa, and Nigeria, contributing two third of the overall funding [15]. We imagine that a similar situation would prevail for COVID-19 research; therefore, it is imperative that African countries actively seek to fund COVID-19 research and other research priorities to drive their research agenda and take ownership.

The majority of the registered studies were registered prospectively, implying high compliance with WHO and ICJME regulations on clinical trial registration [8]. However, this requirement is only applicable to clinical trials and not observational studies. We cannot discern if the prospective/retrospective clinical trial registration field was completed for only clinical trials using the available data.

The epidemiology of COVID-19 is still unraveling, with highlighted knowledge gaps that need to be filled with evidence from robust research [12, 16]. This is more pertinent with the emergence of new strains of the SARS-COV2, such as the delta variant that has implications for effective control of the pandemic [17].

Targeted vaccination of at-risk persons against COVID-19 is ongoing in several African countries. These vaccines were granted conditional approval [18, 19] based on clinical trial data mainly not generated from the African population. Therefore, additional research is needed to monitor their safety and efficacy in this population and ensure they continually maintain a favorable risk-benefit profile. Specifically, targeted phase 4 studies will generate data to support a favorable risk-benefit balance of these interventions.

Phases 4 studies of COVID-19 vaccines would have several benefits. On the one hand, such post-licensure studies will ensure that interventions are recommended based on the best available evidence. On the other hand, the public is reassured, which may improve the uptake of vaccination and other interventions. Similarly, well-conducted observational studies will be needed to understand the drivers and predictors of the disease.

### Limitations of this study

Our study has some limitations. First, we relied on studies available in the ICTRP. While this presents complete data on studies registered considering partner registers that are part of the WHO ICTRP, some studies may have been registered in non-partner registers and therefore omitted from our analysis. Furthermore, we noted that the studies in several primary registries were incomplete or missing certain variables, thus limiting our analysis and potentially biasing our results. Secondly, there is no legal/ethical requirement for prospective registration of observational studies, unlike clinical trials; therefore, we believe that the actual number of observational studies conducted in Africa may be higher than what we found in the ICTRP. Similarly, several studies have demonstrated that many studies are not in compliance with the mandated prospective registration of clinical trials, only registering these studies retrospectively, usually before disseminating the study findings in a peer-reviewed journal. Therefore, we may have missed out on some studies that have not been retrospectively registered in our analysis in this context. Finally, data on the various studies analyzed in this manuscript are from varying clinical trial registers that may have varying data completeness levels, thus affecting our ability to analyze the study findings.

Nevertheless, we provide evidence from a standard repository of clinical trials that provides a landscape of COVID-19 research conducted in Africa to help policymakers and the research community decide future research priorities.

## Recommendation and conclusion

Our study highlights some crucial points. We demonstrate that research on COVID-19 is relatively skewed towards the global north, with only a small proportion of studies conducted in Africa. However, taken together with the rather impressive flurry of global research on the subject, it becomes difficult to declare the number of studies conducted in Africa as being inadequate. We also highlight the varied research areas that have been explored during the COVID-19 pandemic, both regionally and globally. Furthermore, we stress the importance of local leadership of COVID-19 research while leveraging on existing north-south collaboration.

## Data Availability

The data used in this study is publicly accessible from the ICTRP portal.
